# Social determinants of heat-related mortality in England: a time-stratified case-crossover study using primary care records

**DOI:** 10.1136/bmjph-2024-001111

**Published:** 2025-07-30

**Authors:** Ross Thompson, Sari Kovats, Helen Macintyre, Shakoor Hajat, Emer O’Connell

**Affiliations:** 1 NIHR Health Protection Research Unit in Environmental Change and Health, London School of Hygiene & Tropical Medicine, London, UK; 2 Extreme Events and Health Protection, UK Health Security Agency, London, UK; 3 UK Health Security Agency, London, UK; 4 Earth and Environmental Sciences, University of Birmingham, Birmingham, UK

**Keywords:** Public Health, Public Health Practice, Environmental Monitoring, Epidemiology

## Abstract

**Background:**

Despite increases in heat-related deaths in England, there has been limited progress in developing interventions in primary care that identify and target individuals at risk. Lack of understanding of individual-level socioenvironmental risk factors limits development of an evidence-based approach to targeted prevention.

**Objective:**

To identify individual-level non-clinical risk factors for heat-related mortality in England using primary care records and to assess the potential of these socio-environmental factors as effect modifiers for the association between ambient temperature and death.

**Methods:**

A time-stratified case-crossover analysis was undertaken of nine potential risk factors at the individual level and categorised into risk factor subgroups. 430 682 patients with valid records were included in the study population, obtained from the Clinical Practice Research Datalink. Conditional logistic regression was used to characterise associations between temperature and the risk of death on hot days and to investigate the modifying effect of each risk factor.

**Results:**

Older ages, females, ethnic minorities and those living in the most deprived areas all had increased risk of death during periods of heat. An increasing trend in ORs was observed with increasing amounts of alcohol intake and increasing body mass index, excluding the obese-3 group. No differences in risks were observed by marital status or frailty category.

**Conclusions:**

This is the first study in England to assess the role of socioenvironmental factors in modifying heat risk at an individual level. The results provide important evidence on the role of disadvantage in driving the inequitable distribution of climate change impacts, and the need for better socioeconomic data linked to health records. For clinical practice, the findings highlight the importance of incorporating an assessment of individual socioenvironmental circumstances when prioritising patients at highest risk during heat events.

**WHAT IS ALREADY KNOWN ON THIS TOPIC**

- The pathway to ill health during periods of heat is complex and involves a combination of exposures, individual-level risk factors and adaptive capacity of the individual. Population-level epidemiological studies exploring individual-level heat risk have generally used routine mortality and emergency hospitalisation data, which do not contain data on wider social determinants of health, which are known to influence risk.

**WHAT THIS STUDY ADDS**

- We explore socioenvironmental factors modifying heat risk at an individual level using primary care records for the first time in England. We identify ethnicity and deprivation as significant risk-modifying factors in England for the first time, along with unexpected patterns in risk by body mass index and frailty.

**HOW THIS STUDY MIGHT AFFECT RESEARCH, PRACTICE OR POLICY**

- We demonstrate that primary care data can provide powerful insights that have implications for patient management during heat events, highlight the complexity of heat risk and the role of socioenvironmental factors in driving that risk and further underscore the urgency of policy action that is required to address health inequalities observed in heat-associated mortality during heat events in England.

## Introduction

 Heatwaves and high temperatures pose significant risks to health.^1^,^5^ In England, there is an increasing trend in total heat-associated mortality.^6^,^8^ 2022 observed the highest heat mortality value following the first 40°C heatwave and associated level 4 Heat-Health Alert and RED Extreme Heat warning, and resulted in 2985 heat-associated deaths.^8 9^ Following the pan-European heatwave in 2003, many countries and cities introduced Heat-Health Action Plans, which set out a framework to plan for and respond to these adverse weather events.^10^ In 2023, the UK Health Security Agency (UKHSA) launched the Adverse Weather and Health Plan (AWHP),^11^ which aims to prevent avoidable harms to health during adverse weather events, including during periods of increased heat. A key action for health and social care providers recommended in the AWHP is to ‘establish methods to identify, alert and monitor individuals most vulnerable to heat-related illnesses on your caseload’.^10 11^ Evidence suggests that this particular recommendation is not widely implemented^12 13^ and one potential contributing factor is the absence of an evidence-based approach through which healthcare professionals can identify individuals most at risk of dying in a heatwave.^13 14^

The pathway to ill health during periods of heat is complex and involves a combination of exposures, individual-level risk factors and adaptive capacity of the individual. At the population level, at-risk groups include older people, the very young and people with pre-existing medical conditions as well as those whose social, housing or economic circumstances put them at greater risk of harm during periods of heat.^3 9^ However, such broadly defined subgroups along with poorly specified aspects of heat risk do not allow for the highest risk individuals to be identified and targeted for intervention before adverse health effects occur. Population-level epidemiological studies exploring individual-level heat risk have generally used routine mortality and emergency hospitalisation data.^15^,^17^ Where wider determinants of health and heat risk have been investigated, these data have generally been linked to restricted registries^15^ or use a small number of proxy measures where individuals are assigned a relevant category based solely on their geographic location.^18^ While this does provide some evidence on area-level risk factors, such as level of vegetation cover in London,^19^ there are a number of assumptions made, which may miss some of the individual-level context of heat risk.

We have recently explored individual-level clinical risk factors and heat using primary care records in England and highlighted important clinical factors associated with increased risk of death during heatwaves.^20^ While patient record systems in primary care are predominantly used for managing clinical care, they also contain other types of data that are relevant to heat risk. In England, vulnerability assessments undertaken as part of routine primary care practice, such as the electronic frailty index^21^ and the QRISK prediction algorithm,^22^ consider a range of factors in addition to clinical aspects to derive risk scores for frailty and cardiovascular risk, respectively. Therefore, this study aims to identify individual-level socioenvironmental risk factors for heat-related mortality in England using primary care records, and to use these data to estimate the potential effect modification of a range of wider determinants of health. Results from this study will build on previous work and provide foundational evidence for the development of methodologies for effectively identifying individuals at risk of heat-related mortality in England, so that targeted interventions can be deployed.

## Methods

### Study population

As previously described,^20^ linked primary care records, Office for National Statistics (ONS) mortality data and NHS hospitalisation data, at the individual level, were obtained from Clinical Practice Research Datalink (CPRD) Aurum (ID number 21_000621). The outcome of interest was defined as all deaths, which occurred between May and September 2016–2020 using ONS date of death. CPRD Aurum has been shown to be representative of the English population in regards to age structure, sex, deprivation and to a lesser extent geographical spread.^23^

Using the same approach as previous studies, we used primary care data to identify individuals with relevant socioenvironmental health determinants, focusing on records within 2 years of death to accurately reflect the individual’s status at that time.^15 20 24^ If multiple records were available within this period, the one closest to the date of death was used. Nine key individual-level factors were selected based on evidence, plausibility and data availability and are presented in online supplemental table S1. These factors were then categorised further into subgroups (see table 1). Published and bespoke clinical code lists were developed to create risk factor variables for the study sample, and these lists are available in online supplemental table S1.

**Table 1:** Overview of data used in analysis

Variable	Observations	Proportion
All persons	430 682	100.00%
Male	211 651	49.14%
Female	219 029	50.86%
Age	430 682	100.00%
Sub-national regions	430 682	100.00%
The North (NE, NW and Y&H)	113 405	26.33%
Midlands and East (WM, EM, EoE)	102 630	23.83%
London	65 145	15.13%
The South (SW and SE)	149 502	34.71%
Alcohol intake category	99 099	23.01%
Non-drinker	3386	0.79%
Light drinker	4624	1.07%
Moderate drinker	88 000	20.43%
Heavy drinker	3089	0.72%
Ethnicity	45 263	10.51%
White	42 280	9.82%
Black	985	0.23%
Asian	1317	0.31%
Other ethnicity	681	0.16%
Living arrangement	16 874	3.92%
Living alone	11 609	2.70%
Cohabiting	4950	1.15%
Homeless	315	0.07%
Marital status	17 229	4.00%
Single/divorced/widowed	5773	1.34%
Married/has partner	11 456	2.66%
Body mass index category	134 884	31.32%
Underweight	15 602	3.62%
Normal weight	54 742	12.71%
Overweight	37 044	8.60%
Obese 1	17 146	3.98%
Obese 2	6504	1.51%
Obese 3	3864	0.90%
Frailty category (eFI)	83 968	19.50%
Fit	4285	0.99%
Mildly frail	15 805	3.67%
Moderately frail	28 290	6.57%
Severely frail	35 604	8.27%
Index of multiple deprivation	430 682	100.00%
1 (least deprived)	41 757	9.70%
2	41 611	9.66%
3	43 581	10.12%
4	44 693	10.38%
5	40 801	9.47%
6	43 300	10.05%
7	43 157	10.02%
8	40 784	9.47%
9	44 597	10.36%
10 (most deprived)	46 397	10.77%

Total number of practices contributing to sample = 1476.

Mean number of patients per practice = 291.79.

Maximum number of patients per practice = 2400.

Minimum number of patients per practice = 6.

eFI, electronic frailty index; EM, East Midlands; EoE, East of England; Lon, London; NE, Northeast; NW, Northwest; SE, Southeast; SW, Southwest; WM, West Midlands; Y&H, Yorkshire and the Humber.

### Exposure data

Geographical information for individuals in the study was limited to UK government region of their registered primary care practice. To address this, a daily mean population-weighted regional temperature series was generated for the study period using HadUK-grid daily maximum and minimum temperatures^25 26^ to create regional population-weighted temperature series, which were then assigned to each individual based on their GP practice region, as previously described in detail.^20^ A 3-day lag period was calculated to estimate delayed and cumulative effects of temperature exposure, focusing on immediate heat effects.^27^

### Statistical analysis

As with our previous analysis, a time-stratified case-crossover study design was used to examine the association between temperature and mortality.^20^ In this design, temperature on the day of death (event day) is compared with non-event days. The main relationship under investigation is the association between temperature and risk of death on days at specified temperature thresholds using conditional logistic regression. Each individual serves as their own control, which automatically controls for time-invariant confounders like age or gender. As with our previous study, control days were selected following a bidirectional referent selection approach, which is the most common approach for such study designs, to be the same day of the week of the same month of the same year in which the death occurred, resulting in each case having at least three controls, reducing potential for overlap bias.^20 28 29^

As has been outlined in detail previously,^20^ there were three stages to the analysis. First, the association between temperature and mortality was modelled to assess the dose–response relationship between temperature and risk of death across the whole population. This was carried out using natural cubic spline functions, with internal knots determined using the Akaike Information Criterion to define the best model fit, an approach that has previously been reported.^20 30^ From this initial model, temperature thresholds for analysis were derived using the approach used by the UKHSA for defining the ‘low’ impact level (temperature associated with relative risk of 1.1) of the new impact-based heat-health alert system.^31^ The ‘Medium’ impact threshold (temperature associated with a RR of 1.2) was used in sensitivity analysis. The ‘high’ impact threshold was not used due to daily temperatures within the study period not reaching the required 40°C temperatures, as defined by UKHSA. Relative thresholds were derived for the national-level analysis and for subnational-level analysis, used in sensitivity analysis. The reference temperature for the conditional logistic regression was taken as the temperature at which RR equated to 1.0, referred to from here as the reference mortality temperature (RMT). This approach was taken to ensure policy relevance of the analysis.

Second results were then stratified by subpopulation categories to assess effect modification with all results reported as OR with 95% CIs and p values. Finally, a relative effect modification (REM) index was calculated as the specific OR of an individual-level factor compared with a reference category to aid interpretation of OR estimates. All analyses were carried out in Stata Statistical Software: release 17.^32^

### Subnational-level analysis

The analysis was repeated at subnational level to assess potential geographical variations in estimated associations, as previously described.^20^ Subnational-level analysis was carried out using the following regional groups: London; The North (combined North East, North West and Yorkshire and Humber); Midlands and East (combined West Midlands, East Midlands and East of England) and the South (South West and South East). Regions were combined to ensure that the frequency of the events (deaths, heat-health alerts) was sufficient to support the analysis and based on: study population frequencies, the study population distribution compared with the national distribution over the study period, number of Heat-Health Alerts issued over the study period, geographic location and climate.

### Sensitivity analyses

To assess the robustness and generalisability of the results of the analysis, three separate sensitivity analyses were undertaken using the same approach as previous studies.^20^ First, the analysis was repeated using the ‘medium impact’ threshold to assess any differences in the patterns of ORs. Second, we assessed the potential confounding effect of background air pollutants on a restricted number of variables. Particulate matter (PM_10_), ozone (O_3_) and nitrogen dioxide (NO_2_) are all potential confounders of the association between heatwaves and mortality.^33^ Due to data limitations, this analysis was restricted to London using daily mean NO_2_, PM_10_ and O_3_ concentration. Five urban background air quality monitoring sites were selected across London from the London Air Quality Network.^34^ Using daily means for each site, a London-wide daily mean background value was derived for each pollutant and assigned to cases in London. As with temperature, a 0–2-day lag period was also calculated and assigned to each individual.

### Patient and public involvement

The Health Protection Research Unit in Environmental Change and Health has developed a public engagement/involvement group called Public Led and Knowledge Engagement Team (PLANET), which was established in Autumn of 2020. The group has 30 members and approximately 20 attend the regular meetings every 3–4 months to discuss research projects. A session exploring heat risk and use of primary care records to identify those at highest risk were carried out in May 2022 where the aims and objectives were presented to the group for feedback. Results from this and linked studies were presented to the PLANET group in early 2024 at an annual meeting, with the meaning and implications of the results discussed, and potential future research in this area explored.

## Results

430 682 individuals who died over the study period (May to September, 2016–2020) from 1476 primary care practices were included in the analysis. An overview of all individuals with a suitable record for each variable is provided in table 1. Details of exposure data (temperature and air pollutant concentrations) are provided in online supplemental table s2.

### Temperature thresholds

**Figure 1:**
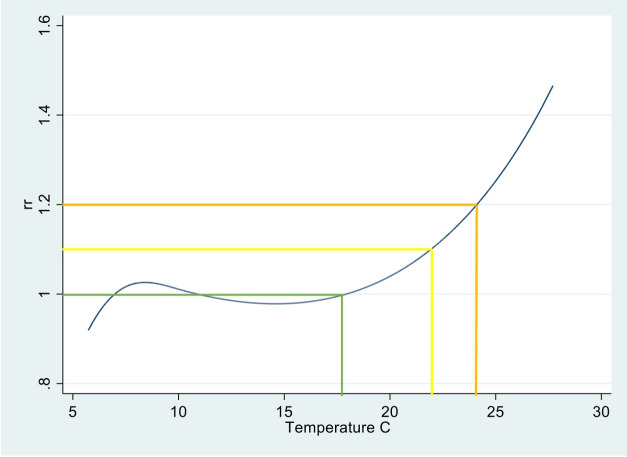
National temperature–mortality relationship plot of relative risk (RR) and mean temperature from which temperature thresholds used in analysis were derived. Green line represents the RMT with a RR=1.0 which equates to about 17°C (rounded to the nearest 0.5°); yellow line indicates the UKHSA defined low impact threshold with a RR of 1.1 which equates to 22°C; amber lines indicate the UKHSA defined medium impact threshold with a RR of 1.2 which equates to 24°C, which was used in sensitivity analysis. RMT, reference mortality temperature; UKHSA, UK Health Security Agency.

Figure 1 illustrates the temperature–mortality relationship derived using the full data series (ie, all individuals within the study population) and the temperature thresholds derived. The policy-relevant thresholds, when rounded to the nearest 0.5°C equate to 17°C (the RMT), 22°C and 24°C. Using these thresholds to identify cases resulted in 13 970 using the ‘low’ impact threshold and 10 187 using the ‘medium’ impact threshold. Temperature thresholds used for subnational level sensitivity analysis are reported in table S3 in online supplemental materials.

### Age, sex, ethnicity

Heat mortality risk was modified by age, sex and ethnicity when comparing the odds of death at the RMT (17°C) and at the ‘low impact’ temperature (22°C). Risk of death during heat episodes increased with age, regardless of size of age groups, however, 95% CIs overlap across age groups with the OR estimates for over 65-year groups relatively consistent. Females have somewhat higher risk than males within the study population, but this was not statistically significant.

**Figure 2:**
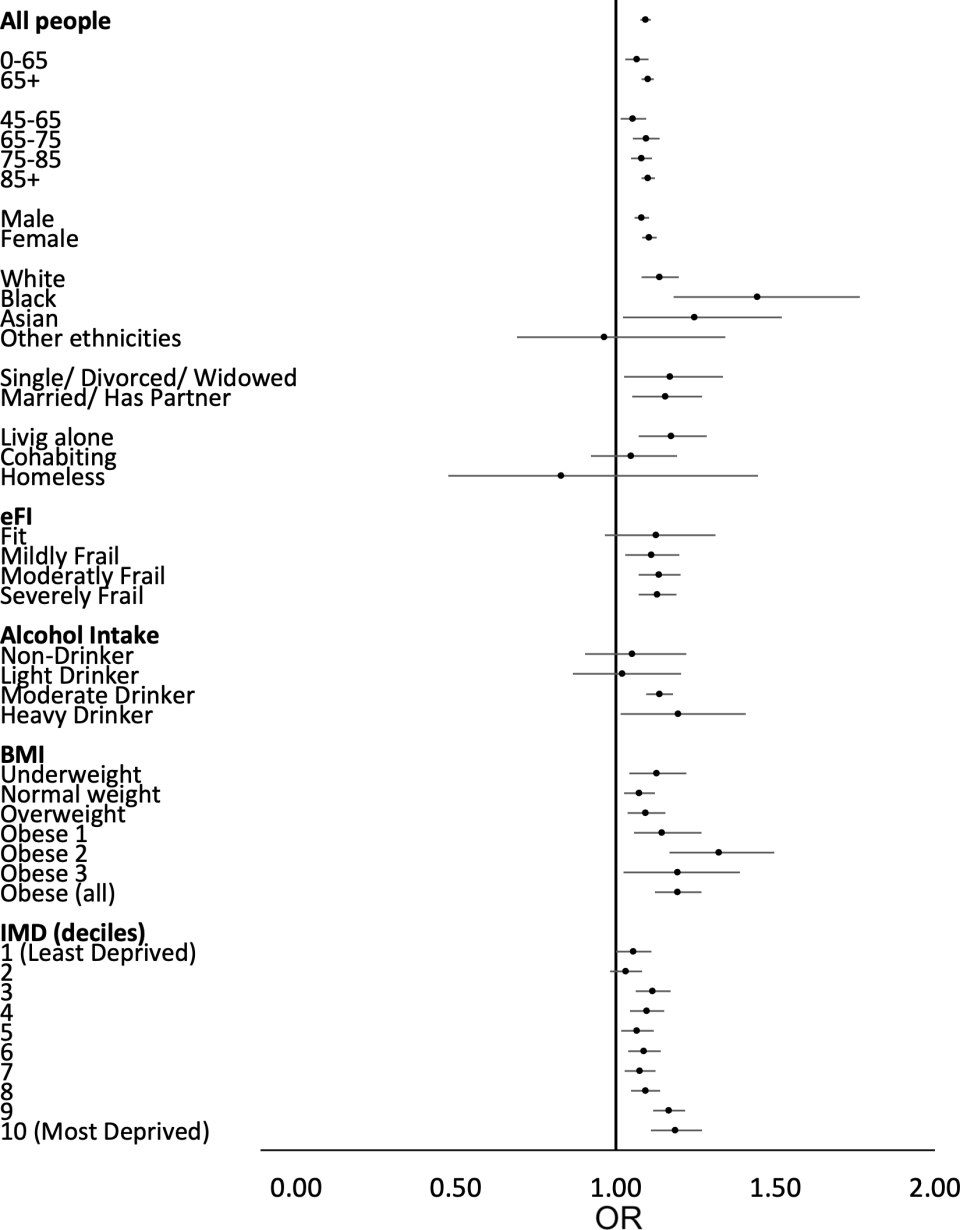
Forest plot showing the estimated OR and 95% CIs by age group, gender, ethnicity, marital status living arrangement, frailty (electronic frailty index), alcohol intake, BMI and deprivation (IMD) using the UKHSA defined HHA low impact thresholds at the national level. BMI, body mass index; eFI, electronic frailty index; IMD, index of multiple deprivation; UKHSA, UK Health Security Agency.

Of the 10.5% of individuals for whom ethnicity was recorded, those of black or Asian ethnicity had substantially higher risk than those who are white, with an REM index of 1.27 for those of black ethnicity and of 1.10 for those with Asian ethnicity (white ethnicity as the reference group) (figure 2 and Table S4 in online supplemental materials).

### Marital status and living arrangements

Risk of death during heat episodes for those who are single, divorced or widowed is slightly higher than those for individuals who are married or who have a partner, but overlapping CIs indicate that there is likely to be little difference in the risk between these groups. There is some evidence that those who are living alone have increased risk of death on hot days compared with those who are cohabiting. Unfortunately, the numbers of individuals categorised as homeless was too small to provide meaningful OR estimates.

### Electronic frailty index

Overall, no trend in risk by electronic frailty index (eFI) category was observed. The similarity of the OR point estimates and overlapping CIs for each category of the eFI indicates no difference in the risk profile across eFI categories (fit, mildly frail, moderately frail and severely frail), see figure 2.

### Alcohol intake

Those classed as heavy drinkers had the highest risk of death during heat episodes compared with the other classes of alcohol intake, with moderate drinkers also having increased risk compared with light and non-drinkers, who appear to have no evidence of an association between mortality and high temperatures.

### Body mass index

Individuals who are categorised as underweight and overweight (including obese 1–3) have increased risk of heat-related death compared with those who are considered normal weight. There is a clear J-shaped trend in ORs by body mass index (BMI) category, except for the highest BMI category, obese 3, where the OR estimate reduces considerably (see figure 2). However, when all obesity subcategories are combined into a single group, the OR of the combined obese group is raised, and this finding is statistically significant.

### Deprivation

Patterns observed in risk of heat-related death by deprivation group highlight that the highest risk in heat-related deaths occurs for those within the two most deprived groups, while those in the least deprived groups have the lowest risk, with the difference between the ORs for the most deprived groups (IMD 9 and 10) with the least deprived groups (IMD 1 and 2) statistically significant. While the relationship does not appear to be linear across all groups, with those living in areas with an IMD value of 3 up to 7 having comparable ORs, there does appear to be a general trend in increasing risk with increasing deprivation score.

### Subnational and sensitivity analysis

In general, the patterns observed for the estimated ORs described above were largely consistent when the analysis was repeated using the medium impact threshold temperatures (online supplemental table S4) and at subnational level (online supplemental table S5). However, the patterns observed for deprivation at the subnational level did not quite match those of the national patterns, and there were two specific differences observed for the London analysis which are notable. First, the OR estimates by age group for London are reasonably constant across all groups, unlike the results from the national-level analysis and other subnational areas where OR estimates generally increase with age (see online supplemental table S5). Second, the difference in OR by sex is particularly pronounced in London using the ‘medium’ impact threshold. When the model for London was adjusted for daily mean concentrations of background PM_10_, NO_2_ and O_3_, OR estimates and patterns were consistent with the unadjusted estimates as can be seen in figure S1 in the online supplemental materials.

## Discussion

This study shows that among the study population, there are clear patterns across subgroup populations for a range of health determinants. This included increasing risk with age, differences in risk by sex, ethnicity, living arrangement, alcohol intake, BMI and deprivation. However, no real differences in risk were observed within or between frailty subgroups, illustrating that this routine measure is not a good proxy for heat risk. We demonstrated that our findings are unlikely to be due to confounding from concurrent exposure to air pollution. We also identified some regional nuances in patterns of risk in London that differ from that of the national picture.

The results of increasing risk for older adults and potential differences in risk by sex align with the population-level epidemiological evidence.^335^,^45^ However, our study identified different patterns in London, where risk was more uniformly distributed across the different age groups. Reasons for this are unclear and likely to be a complex combination of many factors. These may include London’s unique population profile compared with other parts of the UK^46^ and complex migratory patterns of movement into and out of London across age groups.^47^

Observed temperatures are generally higher in London than in other parts of the England, with the additional heat burden of the urban heat island effect, which likely increase exposure further,^48^ via indoor overheating risk.^49^ This risk may be further compounded by reduced capacity for adaptive behaviours to reduce overheating risk, for example, security concerns or through the necessity of income, or by other socioeconomic factors unique to the capital, such as high cost of living^50^ and high rates of household overcrowding.^51^ Analysis of inequality in the UK from 2020 suggests that inequality in London is far higher than in other regions of the UK, with over a quarter of Londoners living in poverty and over 15% in the top 10% of earners nationally.^52^ Recent evidence suggests that, in London, more affluent areas also have more access to green space, which is also linked with cooler urban environments, potentially reducing risk.^19^ The potential complexity of contributing factors to heat risk highlighted here just demonstrates the difficulty of ensuring any interventions that are deployed are both adequately targeted and equitable.

Sex-based differences in the ORs are also noteworthy, with women exhibiting higher ORs than men, a finding that is consistent with previous studies.^53^ Reasons behind these differences remain unclear. There is evidence that there may be physiological differences with thermoregulatory responses to exogenous and endogenous heat loads, including core body temperature variation by sex, sweat volume discrepancies and hormonal influences associated with the menstrual cycle.^54^ However, there are a number of other factors, which may also contribute to this apparent difference in risk, such as social and cultural influences,^4^ and other socioeconomic factors and comorbidities which are more prevalent in older females that may increase their risk as their age.^55 56^ In addition, the age distribution across genders is not uniform, with more females in the higher age groups than males,^46^ and therefore the higher ORs observed may be related to age. It is plausible, however, that all of the above play a role in differences in risk observed here and further demonstrates the complexity of contributing factors to heat risk. More research is required to fully explore this trend and the potential causes for these observed differences by sex, including assessing the differences in risk by sex across age groups. This is particularly important in delivering equitable health and care services and reducing health inequalities.^57^

This is the first study to evidence ethnicity as an important risk factor for death during hot days in England. Black and Asian individuals experienced higher risk of mortality on hot days, contrary to previous UK studies.^3^ However, this result may be the consequence of circumstances and structural racism experienced by ethnic minority groups, which lead to increased health inequalities.^58^ In addition, individuals living in the most deprived areas experienced increased risk of death, while those in the least deprived areas displayed the lowest. This trend aligns with evidence globally^18^ and strengthens emerging evidence that deprivation may be a significant risk factor during heat periods in England.^19^ However, the relationship observed was not consistently linear, therefore caution should be taken when considering deprivation alone as a way of characterising heat risk.

The results for ethnicity and deprivation within this study reflect well-documented evidence on the importance of socioenvironmental factors for health equity.^59^ The domains from which IMD is calculated include income, employment, education, health, crime, barriers to housing and services and living environment.^60^ All of these domains affect an individual’s underlying physical and mental health as well as their capacity to adapt either their environments or behaviours when temperatures increase. The ability to adapt to changing conditions is one of the key domains of heat risk. Existing health disparities will also play an important role with clear evidence that those in the lowest IMD groups have significantly poorer health overall, with shorter healthy life expectancy and higher prevalence of long-term conditions many of which are associated with increased risk during heat events.^61^ In addition, there is evidence that poor housing, both current and in the past, is significantly associated with poor health outcomes^62^ and that disadvantaged households are less likely to have adaptive approaches to maintain cool indoor temperatures.^63^ This is particularly important as our buildings are one of the core mediating factors of the temperatures to which we are exposed. These findings underline the importance of considering social determinants of health in assessing heat-related risks and further strengthen the case for addressing health inequalities as part of wider climate adaptation strategies and policy. The findings illustrate the role of climate justice at a local level, with those experiencing the highest risk contributing the least in terms of greenhouse gas emissions: in the UK, the top 1% of earners average 76.6 tons of CO2 equivalent per capita, compared with 5.6 tons of CO2 equivalent per capita for the bottom 50%.^64^

Results for eFI and BMI analysis suggest there may be subtle distinctions between those who are considered clinically vulnerable and those at-risk of death during periods of heat. There is considerable overlap between factors used to calculate eFI and those identified within the literature associated with increased heat risk.^18^ Therefore, it is plausible to assume that a high eFI score could be a reasonable proxy for heat risk. Similarly, it’s plausible that those in the highest BMI category could be prioritised due to their risk profile from a physiological perspective.^65^ But neither measure was a reliable proxy in this study. For eFI, the ORs align with the estimates for older age groups, which indicates no change in effect, as eFI assessments are only carried out on those aged 65+. This absence of a trend could stem from the fact that moderately frail individuals may be receiving care to limit their transition into the higher category of frailty, while those classed as severely frail will be in receipt of clinical review and management.^66^ In addition, individuals classed as ‘Obese 3’ may be referred to tier 3 wt management—a clinically led approach to reducing an individual’s weight issues.^67^ Thus, the level of care being received by individuals within these groups may lead indirectly or directly to an overall reduction in risk during a heatwave. The distinction between clinical vulnerability and the risk identified in the current study has important implications for how patients are prioritised by clinicians during periods of heat. These findings align with previous studies,^20 24^ which highlighted that individuals at greatest risk are not limited to those with the most severe disease, and that those considered more resilient in general are also at risk during periods of heat.

### Limitations of the study

Geographical resolution of the health data meant that precise exposure assignment for each individual within the study was not possible. However, previous studies^1^ have demonstrated high correlation between temperature monitoring stations within English regions, and that it is possible to characterise exposure well using a regionally representative temperature series. Inconsistencies in primary care consultation records and the specific terms used when recording details by clinicians increase the potential for some relevant records to be missed. However, our systematic approach for identifying relevant records, which included clinical validation, should address this limitation. It is also unlikely that these inconsistencies or missing records are correlated with exposure or outcome, therefore unlikely to be a source of bias. While CPRD is representative of the English population,^23^ it does not have full coverage of England, and is not geographically representative.^23^ Episode analysis of previous heatwaves suggests most heatwave-related deaths occur in the south.^6^,^868^ Therefore, it is not anticipated that this limitation would affect our results significantly.

These limitations are compounded by data challenges in relation to the completeness and representativeness of some primary care records, and therefore caution is required when interpreting these results. For example, only 10.2% of patients in the study had a valid record for ethnicity—the findings of this study suggest that there may be important differences in the risk profile of ethnic minority individuals, but it is difficult to draw strong conclusions as the majority of patients lacked these data, despite ethnicity being one of the indicators of the Quality and Outcomes Framework in the UK in which payments are made to encourage recording of such information.^72^ Similarly, only 3.9% in individuals had a record of living arrangement, with only 0.07% with a record of being homeless. Such small proportions make gaining any insight about this specific group very challenging, despite evidence that this is an at risk group during periods of heat.^73^ An additional obsticle may be that for some variables, such as alcohol intake, assessment may only be applied to those who have problematic habits, and with a high proportion of missing values it is difficult to draw strong conclusions. However, previous studies have demonstrated internal and external validity of the alcohol consumption data where it is recorded.^74^ There are also potential issues with collinearity of some of the wider determinants of health (eg, ethnicity and IMD) that may be important to consider in the development of any risk stratification approach, however that was not the primary objective of this study. We have also highlighted a potential limitation of relying solely on primary care records, or on clinical risk factors alone, for comprehensive heat risk assessment. The importance of the wider determinants in relation to an individual’s heat risk has been well demonstrated; however, as these important data are not routinely collected through electronic records or noted within clinical consultations, it will be difficult to design targeted, evaluated and cost-effective policy interventions in the absence of this foundational evidence. The role of housing (affordable, accessible, healthy) as a core determinant of health and as a mediator of environmental exposures is well defined^75^; therefore, generation and integration of housing data with clinical and non-clinical records should be a priority area for research funders.

## Conclusion

This study has demonstrated that information related to socioenvironmental determinants of heath as recorded within primary care records can provide important insight about an individual’s heat risk. The role of intersecting risks is nuanced and complex, with aspects that are not apparent in the population-level data. A key finding of this study is the subtle difference between clinical vulnerability and risk during periods of heat, which has important implications for the identification and management of priority patients during heatwaves. While these results demonstrate the utility of primary care data when assessing non-clinical risk factors, the completeness of records remains a significant challenge. Our results illustrate the complexity of factors that drive heat-related health outcomes and the necessity for evidence-based approaches for assessing risk that accounts for both the clinical and contextual factors influencing an individual’s overall risk. This study has also indicated the inequitable burden of impacts experienced by those of non-white ethnicity and those with the least adaptive capacity. Climate change will widen health inequalities, and heat-related harms in particular will be an important outcome of compounding inequalities across exposure, clinical vulnerability and wider socioenvironmental disadvantage. The urgency for policy to address these factors is increasing as the climate continues to change and heat events occur more frequently, last longer and are more intense. This research provides foundational evidence for the development of risk management strategies that target those at greatest risk for the deployment of effective interventions. This is an essential step to tackle the increasing trend in heat-related mortality.

## Supplementary material

10.1136/bmjph-2024-001111 online supplemental file 1

## Data Availability

Data may be obtained from a third party and are not publicly available.

## References

[1] Armstrong BG, Chalabi Z, Fenn B (2011). Association of mortality with high temperatures in a temperate climate: England and Wales. J Epidemiol Community Health.

[2] Hajat S, Kovats RS, Atkinson RW (2002). Impact of hot temperatures on death in London: a time series approach. J Epidemiol Community Health.

[3] Hajat S, Kovats RS, Lachowycz K (2007). Heat-related and cold-related deaths in England and Wales: who is at risk?. Occup Environ Med.

[4] Kovats RS, Hajat S (2008). Heat stress and public health: a critical review. Annu Rev Public Health.

[5] Leonardi GS, Hajat S, Kovats RS (2006). Syndromic surveillance use to detect the early effects of heat-waves: an analysis of NHS direct data in England. Soz Praventivmed.

[6] Gov.uk: Public Health (2020). Heatwave mortality monitoring report: 2020. phe heatwave mortality monitoring.

[7] gov.uk: UK Health Securety Agency (2021). Team EEaHP, ed. Heat mortality monitoring.

[8] gov.uk: UK Health Securety Agency (2022). team EEaHP, ed. Heat mortality monitoring.

[9] HM Government (2024). Agency UHS.

[10] WHO (2021). Europe WROf, ed.

[11] HM Government (2023). Agency UHS, ed.

[12] Lorraine Williams BE, Ettelt S, Hajat S (2019). Evaluation of the heatwave plan for england, final report. https://piru.ac.uk/.

[13] Brooks K, Landeg O, Kovats S (2023). Heatwaves, hospitals and health system resilience in England: a qualitative assessment of frontline perspectives from the hot summer of 2019. BMJ Open.

[14] Wilson L, Black D, Veitch C (2011). Heatwaves and the elderly - The role of the GP in reducing morbidity. Aust Fam Physician.

[15] Stafoggia M, Forastiere F, Agostini D (2006). Vulnerability to heat-related mortality: a multicity, population-based, case-crossover analysis. Epidemiology.

[16] Rocklöv J, Forsberg B, Ebi K (2014). Susceptibility to mortality related to temperature and heat and cold wave duration in the population of Stockholm County, Sweden. Glob Health Action.

[17] Schifano P, Cappai G, De Sario M (2009). Susceptibility to heat wave-related mortality: a follow-up study of a cohort of elderly in Rome. Environ Health.

[18] Son J-Y, Liu JC, Bell ML (2019). Temperature-related mortality: a systematic review and investigation of effect modifiers. Environ Res Lett.

[19] Murage P, Kovats S, Sarran C (2020). What individual and neighbourhood-level factors increase the risk of heat-related mortality? A case-crossover study of over 185,000 deaths in London using high-resolution climate datasets. Environ Int.

[20] Thompson R, Kovats S, Hajat S (2024). Identification of individual-level clinical factors associated with increased risk of death during heatwaves: a time-stratified case-crossover study using national primary care records in England. BMJ Public Health.

[21] Clegg A, Bates C, Young J (2016). Development and validation of an electronic frailty index using routine primary care electronic health record data. Age Ageing.

[22] Hippisley-Cox J, Coupland C, Brindle P (2017). Development and validation of QRISK3 risk prediction algorithms to estimate future risk of cardiovascular disease: prospective cohort study. BMJ.

[23] Wolf A, Dedman D, Campbell J (2019). Data resource profile: Clinical Practice Research Datalink (CPRD) Aurum. Int J Epidemiol.

[24] Stafoggia M, Forastiere F, Agostini D (2008). Factors affecting in-hospital heat-related mortality: a multi-city case-crossover analysis. J Epidemiol Community Health.

[25] Hollis D, McCarthy M, Kendon M (2018). HadUK-Grid Gridded and Regional Average Climate Observations for the Uk.

[26] Hollis D, McCarthy M, Kendon M (2019). HadUK‐Grid—A new UK dataset of gridded climate observations. Geoscience Data Journal.

[27] Armstrong B (2006). Models for the Relationship Between Ambient Temperature and Daily Mortality. Epidemiology (Sunnyvale).

[28] Austin H, Flanders WD, Rothman KJ (1989). Bias arising in case-control studies from selection of controls from overlapping groups. Int J Epidemiol.

[29] Tobias A, Kim Y, Madaniyazi L (2024). Time-stratified case-crossover studies for aggregated data in environmental epidemiology: a tutorial. Int J Epidemiol.

[30] Hajat S, Haines A, Sarran C (2017). The effect of ambient temperature on type-2-diabetes: case-crossover analysis of 4+ million GP consultations across England. Environ Health.

[31] Adverse Weather and Health Plan Agency UHS, ed.

[32] StataCorp LLC (2021). Stata statistical software: release 17.

[33] Stafoggia M, Michelozzi P, Schneider A (2023). Joint effect of heat and air pollution on mortality in 620 cities of 36 countries. Environ Int.

[34] London Air (2023). London IC, ed. https://www.londonair.org.uk.

[35] Almeida SP, Casimiro E, Calheiros J (2010). Effects of apparent temperature on daily mortality in Lisbon and Oporto, Portugal. Environ Health.

[36] Breitner S, Wolf K, Devlin RB (2014). Short-term effects of air temperature on mortality and effect modification by air pollution in three cities of Bavaria, Germany: a time-series analysis. Sci Total Environ.

[37] Gasparrini A, Armstrong B, Kovats S (2012). The effect of high temperatures on cause-specific mortality in England and Wales. Occup Environ Med.

[38] Goodman PG, Dockery DW, Clancy L (2004). Cause-specific mortality and the extended effects of particulate pollution and temperature exposure. Environ Health Perspect.

[39] Huynen MM, Martens P, Schram D (2001). The impact of heat waves and cold spells on mortality rates in the Dutch population. Environ Health Perspect.

[40] Iñiguez C, Ballester F, Ferrandiz J (2010). Relation between temperature and mortality in thirteen Spanish cities. Int J Environ Res Public Health.

[41] Gasparrini A, Guo Y, Sera F (2017). Projections of temperature-related excess mortality under climate change scenarios. Lancet Planet Health.

[42] Oudin Åström D, Åström C, Rekker K (2016). High Summer Temperatures and Mortality in Estonia. PLoS ONE.

[43] Pattenden S, Armstrong B, Milojevic A (2010). Ozone, heat and mortality: acute effects in 15 British conurbations. Occup Environ Med.

[44] Ragettli MS, Vicedo-Cabrera AM, Schindler C (2017). Exploring the association between heat and mortality in Switzerland between 1995 and 2013. Environ Res.

[45] Rocklöv J, Forsberg B (2008). The effect of temperature on mortality in Stockholm 1998--2003: a study of lag structures and heatwave effects. Scand J Public Health.

[46] Statistics OoN (2023). UK Population Pyramid Interactive.

[47] Trust for London (2021). London’s poverty profile 2021. https://trustforlondon.org/.

[48] Kolokotroni M, Giridharan R (2008). Urban heat island intensity in London: An investigation of the impact of physical characteristics on changes in outdoor air temperature during summer. Sol Energy.

[49] Kovats SaB R (2021). The Third UK Climate Change Risk Assessment Technical Report.

[50] Padley M (2020). A Minimum Income Standard for London 2019: Centre for Research in Social Policy.

[51] JAaMM AM Evidence Review: Housing and Health Inequalities in London.

[52] Agrawal S, Phillips D (2020). Catching up or Falling behind? Geographical Inequalities in the UK and How They Have Changed in Recent Years Ifs.Org: The Institute for Fiscal Studies.

[53] van Steen Y, Ntarladima A-M, Grobbee R (2019). Sex differences in mortality after heat waves: are elderly women at higher risk?. Int Arch Occup Environ Health.

[54] Kaciuba-Uscilko H, Grucza R (2001). Gender differences in thermoregulation. Curr Opin Clin Nutr Metab Care.

[55] Aggarwal NR, Patel HN, Mehta LS (2018). Sex Differences in Ischemic Heart Disease: Advances, Obstacles, and Next Steps. Circ Cardiovasc Qual Outcomes.

[56] Vlassoff C (2007). Gender differences in determinants and consequences of health and illness. J Health Popul Nutr.

[57] Winchester N (2021). Women’s Health Outcomes: Is There a Gender Gap?.

[58] Gee GC, Ford CL (2011). STRUCTURAL RACISM AND HEALTH INEQUITIES: Old Issues, New Directions. Du Bois Rev.

[59] Gronlund CJ (2014). Racial and socioeconomic disparities in heat-related health effects and their mechanisms: a review. Curr Epidemiol Rep.

[60] Ministry of Housing CaLG (2019). The english indices of deprivation 2019 (IoD2019).

[61] Toby Watt AR (2022). Laurie Rachet-Jacquet REAL Centre: Quantifying Health Inequalities in England.

[62] Marsh A, Gordon D, Heslop P (2000). Housing Deprivation and Health: A Longitudinal Analysis. Hous Stud.

[63] Taylor J, McLeod R, Petrou G (2023). Ten questions concerning residential overheating in Central and Northern Europe. Build Environ.

[64] (2022). https://www.degruyter.com/document/doi/10.4159/9780674276598/html.

[65] Speakman JR (2018). Obesity and thermoregulation. Handb Clin Neurol.

[66] Turner G, Clegg A, British Geriatrics Society (2014). Best practice guidelines for the management of frailty: a British Geriatrics Society, Age UK and Royal College of General Practitioners report. Age Ageing.

[67] Gupta S, Chen M (2023). Medical management of obesity. Clin Med (Lond).

[68] PHE Heatwave Mortality Monitoring (2016). PHE Heatwave Mortality Monitoring: Summer 2016.

[69] PHE Heatwave Mortality Monitoring (2017). PHE Heatwave Mortality Monitoring: Summer 2017.

[70] PHE Heatwave Mortality Monitoring (2018). PHE Heatwave Mortality Monitoring; Summer 2018.

[71] Public Health England (2019). PHE Heatwave Mortality Monitoring: Summer 2019.

[72] Alshamsan R, Lee JT, Majeed A (2012). Effect of a UK pay-for-performance program on ethnic disparities in diabetes outcomes: interrupted time series analysis. Ann Fam Med.

[73] Hajat S, Sarran CE, Bezgrebelna M (2023). Ambient Temperature and Emergency Hospital Admissions in People Experiencing Homelessness: London, United Kingdom, 2011-2019. Am J Public Health.

[74] Mansfield K, Crellin E, Denholm R (2019). Completeness and validity of alcohol recording in general practice within the UK: a cross-sectional study. BMJ Open.

[75] Mwoka M, Biermann O, Ettman CK (2021). Housing as a Social Determinant of Health: Evidence from Singapore, the UK, and Kenya: the 3-D Commission. J Urban Health.

