# Supplementary material for: Social determinants of heat-related mortality in England: a time-stratified case-crossover study using primary care records
Source: BMJ Public Health. 2025 Jul 30;3(2):e001111. doi: 10.1136/bmjph-2024-001111 (PMC12315017; doi:10.1136/bmjph-2024-001111)
Supplement: online supplemental file 1 [file bmjph-3-2-s001.pdf]

**Title: Social determinants of heat related mortality in England – a time-stratified case-crossover study using primary care records**

**Authors:**
Ross Thompson[1,2], Sari Kovats[1], Shakoor Hajat[1], Emer O'Connell[1,3]

**Affiliations**:
[1] NIHR Health Protection Research Unit in Environmental Change and Health, London School of Hygiene and Tropical Medicine, London
[2]Extreme Events and Health Protection Team, UK Health Security Agency, London
[3] Public Health Unit, Greater London Authority

**Contents**

Table S1 - Justification for inclusion of individual level variables, definition of variable and source of clinical codes
Table S2. Description of exposure data series in analysis.
Table S3 Temperature thresholds used in sub-national analysis derived from the temperature mortality relationships.
Table S4 – Full results across both low and medium impact temperature thresholds for national analysis
Table S5 – Table 2 National and sub-national level results for individual level factors with very strong to moderate evidence of increased risk of death during periods of heat equating to at least the low impact threshold and the relative effect modification index (REM).
Figure S1 – Forest plot chart of crude estimates and estimates which have been adjusted for background air pollutant concentrations in London

Table S1. Table outlines each variable included in the analysis, justification for inclusion and information about how each was defined and link to clinical code lists used to extract the data.

| **Variable for analysis** | **Hypothesis to be tested and likely mechanisms** | **Definition of variable** | **Reference for CPRD Aurum code list(s) used to define variables of interest** |
|---|---|---|---|
| Age | Risk of death on a hot day increases with age.<br><br>*Likely mechanism:* linked to reduced thermoregulatory responses; increased likelihood of comorbidities and polypharmacy etc | Individuals categorised in the following groups<br>• <65<br>• 65+<br>• 45-65<br>• 65-75<br>• 75-85<br>• 85+ | Individuals age at death provided within CPRD data. |
| Sex | There is no difference in risk of death by sex.<br><br>*Likely mechanism:* Unclear, but potential for physiological, social and contextual influences | Categorised as male or female. | Individuals sex provided within CPRD data. |
| Ethnicity | Risk of death during a heat episode differs by ethnic group.<br><br>*Likely mechanism:* Unclear but if a difference is observed, it may be more to do with social drivers of inequality rather than a physiological difference. There is little evidence of this in the UK. | Due to low number of records only broad categories are possible.<br><br>• White<br>• Asian<br>• Black<br>• Other ethnic groups | Mathur, R (2021). Risk factor codelist - Ethnicity. [Data Collection]. London School of Hygiene & Tropical Medicine, London, United Kingdom. https://doi.org/10.17037/DATA.00002414. |

| | | | |
|---|---|---|---|
| Marital Status | Individuals who are unmarried/widowed/divorced are at higher risk of death during heatwaves.<br><br>*Likely mechanism:* This is likely to be a proxy for more social vulnerabilities which increased risk of an individual, such as social isolation, but has been identified as a risk factor in Italy. | All terms related to marital status have been included, searching specifically for divorced, widow partner and married.<br><br>Categorical variable<br>• Unmarried/divorced/widowed<br>• Married | Bespoke code list generated for this project by Daniel Omoyeni and Ross Thompson, clinically validated by Luis Baptista Mieiro<br><br>List available on GitHub here:<br>https://github.com/Rossdud/Clinical-code-lists |
| Living arrangement | People living alone or who are homeless are at increased risk of death during heatwaves.<br><br>*Likely mechanism*: several potential mechanisms to consider depending on which group are considered. Homeless individuals are potentially at increased risk due to a range of social vulnerabilities and other underlying issues which may contribute to those individuals living arrangements. Individuals living along may also lack social connections which increase risk during heatwaves. | All terms related to living arrangements have been included. E.g. information recorded about partner, living in temporary accommodation/hostel etc.<br><br>Categorical variable:<br>• Homeless<br>• Living alone<br>• Co-habitation | Bespoke code list generated for this project by Daniel Omoyeni and Ross Thompson, clinically validated by Luis Baptista Mieiro<br><br>List available on GitHub here:<br>https://github.com/Rossdud/Clinical-code-lists |
| Electronic frailty index | 1) Individuals who are assessed as being mildly, moderately, and severely frail during routine frailty assessments are also at increased risk of death during heatwaves.<br>2) Heat risk increases with increasing eFI category | eFI was not calculated for each individual within the study, but they were included and used where a relevant record, therefore this variable is restricted to over 65s only. Some individuals were already classed as fit, mildly frail, moderately frail or severely frail, while others only had an eFI value. | Bespoke code list generated for this project by Daniel Omoyeni and Ross Thompson, clinically validated by Luis Baptista Mieiro<br><br>List available on GitHub here:<br>https://github.com/Rossdud/Clinical-code-lists |

| | | | |
|---|---|---|---|
| | *Likely mechanism*: frailty assessment is a ratio of the number of "deficits" present in a patients records, and is a general measure of vulnerability. Therefore it is plausible that eFI may also indicate heat risk | These were categorised and combined into one group as outlined below.<br><br>Categorical variable:<br>• Fit - 0-0.12<br>• Mildly Frail = 0.12 to 0.24<br>• Moderately Frail = 0.24 to 0.36<br>• Severely Frail = 0.36+ | |
| Alcohol abuse and misuse | Risk of death on hot days are higher for individuals who drink a lot compared to individuals who do not.<br><br>*Likely mechanism*: dehydration; for heavy drinkers lack of ability to adapt ones own behaviour or environment | Within GP records either the amount of alcohol consumed regularly is recorded, or an individual is categorised as a non-drinker, light drinker, moderate drinker or heavy drinker. Previous studies suggest that this categorisation is complete enough within CPRD to justify using this information in analysis.<br><br>Categorical data<br>• non-drinker<br>• Light drinker<br>• Moderate drinker<br>• Heavy drinker | Bespoke code list generated for this project by Daniel Omoyeni and Ross Thompson, clinically validated by Luis Baptista Mieiro<br><br>List available on GitHub here:<br>https://github.com/Rossdud/Clinical-code-lists |
| Body mass index (BMI) | Risk of death is higher for individuals with higher BMI during periods of heat.<br><br>*Likely mechanism:* due to higher body mass, the individuals organs are likely to need to work harder (higher strain on the heart for example) than for those with lower body mass | Code list identifies all terms within Aurum to extract either direct measurements of BMI recorded in patient clinical records or extract weight and height measurements to allow BMI to be easily calculated.<br><br>BMI value measurements for individuals have been categorised in line with NHS guidelines.<br>• Underweight - <18.5 kg/m2 | Forbes, H and Carreira, H (2021). Risk factor codelist - Body Mass Index (BMI). [Data Collection]. London School of Hygiene & Tropical Medicine, London, United Kingdom. https://doi.org/10.17037/DATA.00002413. |

| | | | |
|---|---|---|---|
| | | • Healthy 18.5 to 24.9 kg/m2<br>• Overweight - 25 to 29.9 kg/m2<br>• Obese 1 - 30 to 34.9 kg/m2<br>• Obese 2 - 35 39.9 kg/m2<br>• Obese 3 - 40-80 kg/m2 | |
| Deprivation | Risk of death on a hot day is higher for those considered most deprived<br><br>*Likely mechanism*: Unclear, but likely related to all domains of deprivation (income, employment, education, health, crime, barriers to housing and services and living environment) | Index of Multiple Deprivation (IMD) Deciles:<br>• 1 = least deprived<br>• 10 = most deprived | IMD provided by CPRD based on the individuals GP practise address |

Table S2. Description of exposure data series in analysis.

| Variable | Observations | Proportion | Mean | Std. dev. | Min | Max |
|---|---|---|---|---|---|---|
| Temperature (°C) *(England)* | 430,682 | 100.00% | 15.7 | 3.3 | 5.7 | 27.7 |
| London | 65,145 | 26.33% | 17.1 | 3.5 | 7.7 | 27.7 |
| The South *(SW & SE)* | 149,502 | 23.83% | 15.9 | 3.1 | 6.7 | 25.6 |
| Midlands and East *(WM, EM, EoE)* | 102,630 | 15.13% | 15.6 | 3.3 | 6.1 | 26.4 |
| The North *(NE, NW & Y&H)* | 113,405 | 34.71% | 14.9 | 3.0 | 5.7 | 24.8 |
| Ozone (ug/m$^3$) *(London)* | 65,145 | 15.13% | 48.9 | 16.1 | 11 | 126.7 |
| $NO_2$ (ug/m$^3$) *(London)* | 65,145 | 15.13% | 21.9 | 9.8 | 3.5 | 61.4 |
| $PM_{10}$ (ug/m$^3$) *(London)* | 65,145 | 15.13% | 16.7 | 7.4 | 6 | 47.7 |

NW = Northwest; NE = Northeast; Y&H = Yorkshire and the Humber; WM = West Midlands; EM = East Midlands; EoE = East of England; Lon = London; SE = Southeast; SW = Southwest

Table S3 Temperature thresholds used in sub-national analysis derived from the temperature mortality relationships.

| Sub-national area | MMT = RR=1 | Low =RR-1.1 |
|---|---|---|
| London | 17 | 22 |
| The South *(SW & SE)* | 17 | 21.5 |
| Midlands and East *(WM, EM, EoE)* | 17 | 22 |
| The North *(NE, NW & Y&H)* | 16 | 21.5 |

NW = Northwest; NE = Northeast; Y&H = Yorkshire and the Humber; WM = West Midlands; EM = East Midlands; EoE = East of England; Lon = London; SE = Southeast; SW = Southwest

Table S4 OR estimates and 95% CI and p-values for all clinical individual-level risk factors investigated, for the whole population using both the Low Impact threshold (temperature associated with RR of 1.1) and Medium Impact threshold (RR of 1.2). In addition, the Relative Effect Modification index (REM) us also reported for each variable investigated.

| Variable | Low Impact Threshold | | | Medium Impact Threshold | | |
|---|---|---|---|---|---|---|
| | OR (95%CI) | p-value | REM | OR (95%CI) | p-value | REM |
| **All people** | 1.09 (1.08 to 1.11) | <0.001 | 1.00 | 1.2 (1.16 to 1.25) | <0.001 | 1.00 |
| **Age** | | | | | | |
| *0-65* | 1.07 (1.03 to 1.1) | <0.001 | 0.98 | 1.14 (1.05 to 1.24) | 0.001 | 0.95 |
| *65+* | 1.1 (1.08 to 1.12) | <0.001 | 1.01 | 1.22 (1.17 to 1.27) | <0.001 | 1.01 |
| *45-65* | 1.05 (1.01 to 1.1) | 0.007 | 0.96 | 1.13 (1.04 to 1.24) | 0.006 | 0.94 |
| *65-75* | 1.09 (1.05 to 1.14) | <0.001 | 1.00 | 1.24 (1.13 to 1.36) | <0.001 | 1.03 |
| *75-85* | 1.08 (1.05 to 1.11) | <0.001 | 0.99 | 1.17 (1.09 to 1.26) | <0.001 | 0.97 |
| *85+* | 1.1 (1.08 to 1.12) | <0.001 | 1.01 | 1.21 (1.16 to 1.27) | <0.001 | 1.01 |
| **Sex** | | | | | | |
| *Male* | 1.08 (1.06 to 1.1) | <0.001 | 1.00 | 1.18 (1.12 to 1.24) | <0.001 | 1.00 |

| | | | | | | |
|---|---|---|---|---|---|---|
| *Female* | 1.1 (1.08 to 1.13) | <0.001 | 1.02 | 1.23 (1.17 to 1.29) | <0.001 | 1.04 |
| **Ethnicity** | | | | | | |
| *White* | 1.14 (1.08 to 1.2) | <0.001 | 1.00 | 1.42 (1.26 to 1.61) | <0.001 | 1.00 |
| *Black* | 1.44 (1.18 to 1.76) | <0.001 | 1.27 | 1.67 (1.1 to 2.53) | 0.017 | 1.17 |
| *Asian* | 1.25 (1.02 to 1.52) | 0.030 | 1.10 | 1.64 (1.02 to 2.63) | 0.042 | 1.15 |
| *Other ethnicities* | 0.96 (0.69 to 1.34) | 0.827 | 0.85 | 0.62 (0.25 to 1.53) | 0.299 | 0.44 |
| **Marital status** | | | | | | |
| *Single/ Divorced/ Widowed* | 1.17 (1.03 to 1.34) | 0.020 | 1.00 | 1.46 (1.06 to 2.01) | 0.022 | 1.00 |
| *Married/ Has Partner* | 1.16 (1.05 to 1.27) | 0.003 | 0.99 | 1.35 (1.07 to 1.71) | 0.011 | 0.93 |
| **Living arrangement** | | | | | | |
| *Living alone* | 1.17 (1.07 to 1.29) | 0.001 | 1.00 | 1.29 (1.04 to 1.62) | 0.023 | 1.00 |
| *Cohabiting* | 1.05 (0.92 to 1.19) | 0.467 | 0.89 | 1.07 (0.8 to 1.44) | 0.647 | 0.83 |
| *Homeless* | 0.83 (0.48 to 1.45) | 0.509 | 0.71 | 0.51 (0.06 to 4.15) | 0.525 | 0.39 |
| **Electronic Frailty Index (eFI)** | | | | | | |
| *Fit* | 1.13 (0.97 to 1.31) | 0.128 | 1.00 | 1.3 (0.92 to 1.85) | 0.142 | 1.00 |
| *Mildly Frail* | 1.11 (1.03 to 1.2) | 0.007 | 0.99 | 1.24 (1.04 to 1.47) | 0.017 | 0.95 |
| *Moderately Frail* | 1.13 (1.07 to 1.2) | <0.001 | 1.01 | 1.37 (1.2 to 1.56) | <0.001 | 1.05 |
| *Severely Frail* | 1.13 (1.07 to 1.19) | <0.001 | 1.00 | 1.25 (1.11 to 1.42) | <0.001 | 0.96 |
| **Alcohol Intake** | | | | | | |
| *Non-Drinker* | 1.05 (0.9 to 1.22) | 0.518 | 1.00 | 1.18 (0.84 to 1.64) | 0.337 | 1.00 |
| *Light Drinker* | 1.02 (0.87 to 1.21) | 0.803 | 0.97 | 1 (0.66 to 1.5) | 0.993 | 0.85 |
| *Moderate Drinker* | 1.14 (1.1 to 1.18) | <0.001 | 1.08 | 1.3 (1.19 to 1.42) | <0.001 | 1.10 |
| *Heavy Drinker* | 1.2 (1.02 to 1.41) | 0.032 | 1.14 | 1.5 (0.99 to 2.26) | 0.055 | 1.27 |
| **BMI** | | | | | | |
| *Underweight* | 1.13 (1.04 to 1.22) | 0.003 | 1.05 | 1.28 (1.07 to 1.54) | 0.008 | 1.15 |
| *Normal weight* | 1.07 (1.03 to 1.12) | 0.002 | 1.00 | 1.11 (0.99 to 1.24) | 0.064 | 1.00 |
| *Overweight* | 1.09 (1.04 to 1.15) | 0.001 | 1.02 | 1.21 (1.06 to 1.37) | 0.005 | 1.08 |
| *Obese 1* | 1.14 (1.06 to 1.27) | 0.001 | 1.07 | 1.51 (1.31 to 1.75) | <0.001 | 1.36 |
| *Obese 2* | 1.32 (1.17 to 1.5) | <0.001 | 1.23 | 1.35 (1.12 to 1.62) | 0.002 | 1.21 |
| *Obese 3* | 1.19 (1.02 to 1.39) | 0.023 | 1.11 | 1.99 (1.47 to 2.71) | <0.001 | 1.79 |
| *Obese (all)* | 1.19 (1.12 to 1.27) | <0.001 | 1.11 | 1.5 (1.01 to 2.22) | 0.046 | 1.35 |
| **Index of Multiple Deprivation (IMD)** | | | | | | |
| 1 (Least Deprived) | 1.05 (1 to 1.11) | 0.048 | 1.00 | 1.19 (1.03 to 1.37) | 0.018 | 1.00 |
| 2 | 1.03 (0.98 to 1.08) | 0.221 | 0.98 | 1.02 (0.9 to 1.16) | 0.733 | 0.86 |
| 3 | 1.12 (1.06 to 1.17) | <0.001 | 1.06 | 1.26 (1.12 to 1.43) | <0.001 | 1.06 |
| 4 | 1.1 (1.04 to 1.15) | <0.001 | 1.04 | 1.17 (1.04 to 1.32) | 0.01 | 0.98 |
| 5 | 1.07 (1.02 to 1.12) | 0.008 | 1.01 | 1.18 (1.05 to 1.32) | 0.004 | 0.99 |
| 6 | 1.09 (1.04 to 1.14) | <0.001 | 1.03 | 1.16 (1.04 to 1.3) | 0.008 | 0.98 |
| 7 | 1.08 (1.03 to 1.12) | 0.002 | 1.02 | 1.2 (1.08 to 1.33) | 0.001 | 1.01 |
| 8 | 1.09 (1.05 to 1.14) | <0.001 | 1.04 | 1.17 (1.07 to 1.28) | 0.001 | 0.98 |
| 9 | 1.17 (1.12 to 1.22) | <0.001 | 1.11 | 1.33 (1.21 to 1.47) | <0.001 | 1.12 |
| 10 (Most Deprived) | 1.19 (1.11 to 1.27) | <0.001 | 1.13 | 1.53 (1.28 to 1.82) | <0.001 | 1.29 |

*Table S5 National and sub-national level results for individual level factors with very strong to moderate evidence of increased risk of death during periods of heat equating to at least the low impact threshold and the relative effect modification index (REM).*

| Variable | National | | London | | The South | | Mids and East | | The North | |
|---|---|---|---|---|---|---|---|---|---|---|
| | OR (95% CI) | REM | OR (95% CI) | REM | OR (95% CI) | REM | OR (95% CI) | REM | OR (95% CI) | REM |
| All people* | **1.09 (1.08 to 1.11)** | **1.00** | **1.09 (1.07 to 1.11)** | **1.00** | **1.09 (1.07 to 1.11)** | **1.00** | **1.07 (1.03 to 1.11)** | **1.00** | **1.17 (1.09 to 1.25)** | **1.00** |
| 0-65 | **1.07 (1.03 to 1.10)** | **1.00** | **1.09 (1.04 to 1.13)** | **1.00** | 0.98 (0.93 to 1.04) | 0.90 | **1.12 (1.01 to 1.24)** | **1.00** | 0.99 (0.85 to 1.16) | 0.85 |
| 65+ | **1.10 (1.08 to 1.12)** | **1.03** | **1.09 (1.07 to 1.11)** | **1.00** | **1.11 (1.08 to 1.13)** | **1.13** | **1.06 (1.01 to 1.11)** | **0.95** | **1.21 (1.12 to 1.30)** | **1.03** |
| 45-65 | **1.05 (1.01 to 1.10)** | **1.00** | **1.09 (1.04 to 1.14)** | **1.00** | 0.97 (0.91 to 1.03) | 1.00 | 1.08 (0.96 to 1.21) | 1.00 | 0.98 (0.83 to 1.17) | 0.84 |
| 65-75 | **1.09 (1.05 to 1.14)** | **1.04** | **1.09 (1.04 to 1.14)** | **1.00** | **1.08 (1.02 to 1.14)** | **1.11** | **1.13 (1.02 to 1.25)** | **1.05** | 1.14 (0.96 to 1.34) | 0.97 |
| 75-85 | **1.08 (1.05 to 1.11)** | **1.02** | **1.08 (1.04 to 1.12)** | **0.99** | **1.07 (1.03 to 1.12)** | **1.11** | 1.03 (0.96 to 1.11) | 0.96 | **1.26 (1.11 to 1.42)** | **1.08** |
| 85+ | **1.10 (1.08 to 1.12)** | **1.04** | **1.09 (1.06 to 1.12)** | **1.00** | **1.11 (1.09 to 1.14)** | **1.15** | **1.04 (1.00 to 1.10)** | **0.97** | **1.23 (1.13 to 1.34)** | **1.05** |
| Sex | | | | | | | | | | |
| *Male** | **1.08 (1.06 to 1.10)** | **1.00** | **1.08 (1.05 to 1.12)** | **1.00** | **1.07 (1.04 to 1.10)** | **1.00** | 1.05 (0.99 to 1.11) | 1.00 | **1.12 (1.01 to 1.23)** | **0.96** |
| *Female* | **1.10 (1.08 to 1.13)** | **1.02** | **1.09 (1.06 to 1.13)** | **1.01** | **1.11 (1.08 to 1.14)** | **1.04** | **1.08 (1.03 to 1.14)** | **1.03** | **1.22 (1.11 to 1.34)** | **1.04** |
| Ethnicity | | | | | | | | | | |
| *White** | **1.14 (1.08 to 1.20)** | **1.00** | **1.14 (1.06 to 1.22)** | **1.00** | - | | - | | - | |
| *Black* | **1.44 (1.18 to 1.76)** | **1.27** | **1.45 (1.19 to 1.78)** | **1.28** | - | | - | | - | |
| *Asian* | **1.25 (1.02 to 1.52)** | **1.10** | **1.25 (1.01 to 1.55)** | **1.10** | - | | - | | - | |
| *Other* | 0.96 (0.69 to 1.34) | 0.85 | 0.84 (0.57 to 1.25) | 0.74 | - | | - | | - | |
| IMD (deciles) | | | | | | | | | | |
| *1* (least)* | **1.05 (1.00 to 1.11)** | **1.00** | 0.99 (0.88 to 1.10) | 1.00 | **1.08 (1.03 to 1.14)** | **1.00** | 1.11 (0.99 to 1.24) | 1.00 | 1.17 (0.91 to 1.49) | 1.00 |
| *2* | 1.03 (0.98 to 1.08) | 0.98 | 1.06 (0.98 to 1.14) | 1.07 | **1.08 (1.02 to 1.14)** | **0.99** | 0.96 (0.85 to 1.09) | 0.87 | 1.18 (0.91 to 1.51) | 1.01 |
| *3* | **1.12 (1.06 to 1.17)** | **1.06** | **1.09 (1.02 to 1.17)** | **1.11** | 1.05 (0.99 to 1.12) | 0.97 | **1.19 (1.06 to 1.33)** | **1.07** | **1.28 (1.02 to 1.61)** | **1.10** |
| *4* | **1.10 (1.04 to 1.15)** | **1.04** | **1.08 (1.01 to 1.16)** | **1.10** | **1.11 (1.05 to 1.19)** | **1.03** | 1.03 (0.92 to 1.16) | 0.93 | **1.53 (1.23 to 1.89)** | **1.31** |
| *5* | **1.07 (1.02 to 1.12)** | **1.01** | 1.04 (0.97 to 1.10) | 1.05 | **1.10 (1.02 to 1.17)** | **1.01** | 1.04 (0.92 to 1.17) | 0.94 | 1.02 (0.80 to 1.31) | 0.88 |
| *6* | **1.09 (1.04 to 1.14)** | **1.03** | **1.09 (1.02 to 1.15)** | **1.10** | **1.10 (1.02 to 1.17)** | **1.01** | 1.07 (0.94 to 1.20) | 0.96 | 1.12 (0.87 to 1.44) | 0.96 |
| *7* | **1.08 (1.03 to 1.12)** | **1.02** | **1.07 (1.02 to 1.13)** | **1.09** | **1.10 (1.03 to 1.18)** | **1.02** | 0.94 (0.82 to 1.08) | 0.85 | **1.35 (1.07 to 1.71)** | **1.16** |
| *8* | **1.09 (1.05 to 1.14)** | **1.04** | **1.09 (1.04 to 1.14)** | **1.10** | **1.15 (1.05 to 1.25)** | **1.06** | 1.07 (0.92 to 1.24) | 0.97 | 0.99 (0.80 to 1.24) | 0.85 |
| *9* | **1.17 (1.11 to 1.22)** | **1.11** | **1.17 (1.11 to 1.23)** | **1.19** | **1.13 (1.04 to 1.23)** | **1.04** | 1.07 (0.92 to 1.24) | 0.96 | **1.30 (1.06 to 1.58)** | **1.11** |
| *10 (most)* | **1.19 (1.11 to 1.27)** | **1.13** | **1.20 (1.09 to 1.33)** | **1.22** | 1.02 (0.91 to 1.14) | 0.94 | **1.18 (1.02 to 1.35)** | **1.06** | 1.03 (0.89 to 1.20) | 0.89 |
| Marital Status | | | | | | | | | | |
| *Single/ Divorced/ Widowed** | **1.17 (1.03 to 1.34)** | **1.00** | **1.22 (1.03 to 1.44)** | **1.00** | 1.10 (0.92 to 1.32) | 1.00 | 0.95 (0.61 to 1.48) | 1.02 | 1.23 (0.70 to 2.16) | 0.92 |
| *Married/ Has Partner* | **1.16 (1.05 to 1.27)** | **0.99** | **1.15 (1.02 to 1.30)** | **0.94** | **1.14 (1.01 to 1.28)** | **1.03** | 0.93 (0.69 to 1.26) | 1.00 | 1.35 (0.82 to 2.20) | 1.00 |
| Living Arrangement | | | | | | | | | | |
| *Living alone** | **1.17 (1.07 to 1.29)** | **1.00** | **1.13 (1.00 to 1.28)** | **1.00** | **1.20 (1.06 to 1.36)** | **1.00** | 1.05 (0.82 to 1.35) | 1.00 | **1.43 (1.01 to 2.02)** | **1.00** |
| *Cohabiting* | 1.05 (0.92 to 1.19) | 0.89 | 1.07 (0.92 to 1.24) | 0.94 | 0.82 (0.63 to 1.05) | 0.68 | 1.08 (0.70 to 1.68) | 1.03 | 1.17 (0.60 to 2.26) | 0.82 |

| | | | | | | | | | | |
|---|---|---|---|---|---|---|---|---|---|---|
| *Homeless* | 0.83 (0.48 to 1.45) | 0.71 | 0.93 (0.53 to 1.64) | 0.82 | - | - | - | - | - | - |
| BMI Category | | | | | | | | | | |
| *Underweight* | **1.13 (1.04 to 1.22)** | **1.05** | 1.09 (0.98 to 1.21) | 1.01 | 1.09 (0.97 to 1.23) | 0.98 | **1.23 (1.02 to 1.48)** | **1.26** | 1.38 (0.99 to 1.94) | 1.38 |
| *Normal weight** | **1.07 (1.03 to 1.12)** | **1.00** | **1.08 (1.02 to 1.15)** | **1.00** | **1.11 (1.05 to 1.18)** | **1.00** | 0.97 (0.97 to 1.09) | 1.00 | 1.01 (0.84 to 1.21) | 1.00 |
| *Overweight* | **1.09 (1.04 to 1.15)** | **1.02** | **1.09 (1.01 to 1.17)** | **1.01** | **1.09 (1.01 to 1.17)** | **0.98** | 1.05 (0.92 to 1.21) | 1.08 | 1.11 (0.90 to 1.38) | 1.11 |
| *Obese 1* | **1.14 (1.06 to 1.27)** | **1.07** | **1.14 (1.02 to 1.27)** | **1.05** | 1.02 (0.91 to 1.14) | 0.92 | **1.35 (1.14 to 1.61)** | **1.39** | 1.25 (0.92 to 1.71) | 1.24 |
| *Obese 2* | **1.32 (1.17 to 1.50)** | **1.23** | **1.31 (1.10 to 1.57)** | **1.21** | **1.17 (1.00 to 1.37)** | **1.05** | 1.26 (0.93 to 1.71) | 1.30 | **1.79 (1.07 to 3.00)** | **1.78** |
| *Obese 3* | **1.19 (1.02 to 1.39)** | **1.11** | **1.30 (1.06 to 1.59)** | **1.20** | 1.10 (0.87 to 1.40) | 0.99 | 0.97 (0.66 to 1.44) | 1.00 | 0.74 (0.35 to 1.53) | 0.73 |
| *Obese (all)* | **1.19 (1.12 to 1.27)** | **1.11** | **1.21 (1.11 to 1.31)** | **1.12** | 1.07 (0.98 to 1.16) | 0.96 | **1.26 (1.10 to 1.45)** | **1.29** | 1.26 (0.99 to 1.62) | 1.26 |

Bold values indicate estimates with moderate to very strong evidence (p<0.05) that individuals with a valid primary care record have an increased odds of death on hot days when a Low impact HHA is likely to be issued by UKHSA

* Indicates the reference group for calculation of the REM index value

A dash (-) indicates where numbers were too small to provide an OR estimate for specific sub-groups

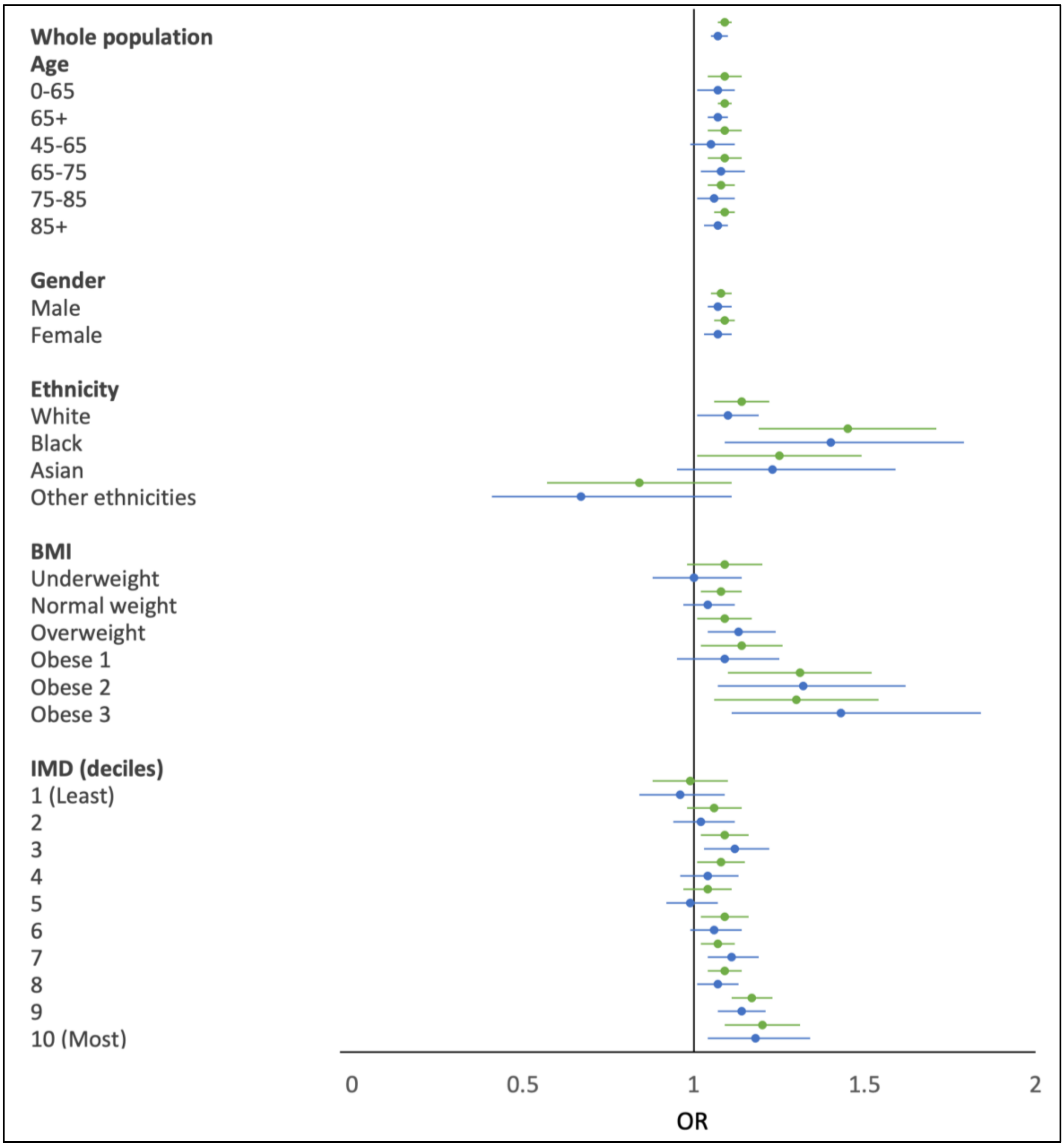


*Figure S1 Forest plot of OR estimates and 95%CIs by age, sex, ethnicity, BMI and IMD categories in London. Green points represent the crude estimates and blue points represent the adjusted estimates for mean daily concentrations of $O_3$, $PM_{10}$ and $NO_2$.*
